# Kefir peptides alleviate high-fat diet-induced atherosclerosis by attenuating macrophage accumulation and oxidative stress in *ApoE* knockout mice

**DOI:** 10.1038/s41598-020-65782-8

**Published:** 2020-05-29

**Authors:** Min-Che Tung, Ying-Wei Lan, Hsin-Han Li, Hsiao-Ling Chen, Sheng-Yi Chen, Yu-Hsuan Chen, Chi-Chien Lin, Min-Yu Tu, Chuan-Mu Chen

**Affiliations:** 1 Department of Life Sciences, and Ph.D. Program in Translational Medicine, National Chung Hsing University, Taichung, 402 Taiwan; 2 Department of Surgery, Tungs’ Taichung Metro Harbor Hospital, Taichung, 435 Taiwan; 3 Graduate Institute of Clinical Medicine, Taipei Medical University, Taipei, 110 Taiwan; 4 Department of Bioresources, Da-Yeh University, Changhwa, 515 Taiwan; 5 Institute of Biomedical Sciences, National Chung Hsing University, Taichung, 402 Taiwan; 6 Aviation Physiology Research Laboratory, Kaohsiung Armed Forces General Hospital Gangshan Branch, Kaohsiung, 820 Taiwan; 7 Department of Biomedical Engineering, Hungkuang University, Taichung, 433 Taiwan; 8 College of Health and Nursing, Meiho University, Pingtung, 912 Taiwan; 9 The iEGG and Animal Biotechnology Center, and the Rong Hsing Research Center for Translational Medicine, National Chung Hsing University, Taichung, 402 Taiwan

**Keywords:** Inflammasome, Atherosclerosis

## Abstract

In the past decade, the high morbidity and mortality of atherosclerotic disease have been prevalent worldwide. High-fat food consumption has been suggested to be an overarching factor for atherosclerosis incidence. This study aims to investigate the effects of kefir peptides on high-fat diet (HFD)-induced atherosclerosis in apolipoprotein E knockout (*ApoE*^*−/−*^) mice. 7-week old male *ApoE*^*−/−*^ and normal C57BL/6 mice were randomly divided into five groups (n = 8). Atherosclerotic lesion development in *ApoE*^*−/−*^ mice was established after fed the HFD for 12 weeks compared to standard chow diet (SCD)-fed C57BL/6 and *ApoE*^*−/−*^ control groups. Kefir peptides oral administration significantly improved atherosclerotic lesion development by protecting against endothelial dysfunction, decreasing oxidative stress, reducing aortic lipid deposition, attenuating macrophage accumulation, and suppressing the inflammatory immune response compared with the HFD/*ApoE*^*−/−*^ mock group. Moreover, the high dose of kefir peptides substantially inhibited aortic fibrosis and restored the fibrosis in the aorta root close to that observed in the C57BL/6 normal control group. Our findings show, for the first time, anti-atherosclerotic progression via kefir peptides consumption in HFD-fed *ApoE*^*−/−*^ mice. The profitable effects of kefir peptides provide new perspectives for its use as an anti-atherosclerotic agent in the preventive medicine.

## Introduction

The World Health Organization (WHO) suggests that cardiovascular diseases (CVDs) are the primary cause of mortality, and considerably more individuals die annually from CVDs than from any other cause globally. Atherosclerosis is known as the major cause of CVDs. The pivotal initiators involved in atherosclerosis development are enhanced levels of low-density lipoprotein (LDL) cholesterol in the circulation, vascular reactive oxygen species (ROS) generation, and inflammation^1^. It has been suggested that inflammation plays a fundamental role in CVDs and atherosclerotic lesion progression^2^. In early atherosclerotic lesions, the accumulation of foam cells leads to fatty streak formation. Immune cells and vascular smooth muscle cells (VSMCs) accumulate in the subendothelial layer of the artery wall^3,4^. Various inflammatory cells, including neutrophils, macrophages, and lymphocytes, are involved in atherosclerosis progression; however, macrophages were reported as the first inflammatory cell associated with atherosclerosis and predominantly present within atherosclerotic vessels^5–7^. With time, fatty streaks grow and change into semimature atherosclerotic plaques; macrophages are subsequently recruited into the area by abnormal endothelium to develop atherosclerotic plaques, which is accompanied by endothelial expression of adhesion molecules, particularly vascular cell adhesion molecule-1 (VCAM-1) and intercellular adhesion molecule-1 (ICAM-1)^5,6^.

Macrophages constitute the majority of atherosclerosis progressions by evolving the plaque instability and subsequent crack of an atherosclerotic plaque, thus resulting in thrombus formation and remodeling^1,8,9^. Furthermore, the generation of ROS in response to LDL oxidation has been shown to be regulated by macrophages^10,11^. The uptake of cholesterol crystals, which are present in atherosclerotic plaques, by macrophages may induce lysosomal destabilization, protease release and ROS production into the cytosol to activate nucleotide binding domain leucine-rich repeat-containing receptor (NLR)-pyrin domain containing 3 (NLRP3) inflammasome, which lead to the operation and secretion of the cytokine IL-1β^12^. Moreover, evidence indicates that macrophages have a strong effect on thrombosis formation. Atherosclerotic endothelium damage results in the reduction of endothelial NO synthase (eNOS) combined with an impaired release of NO and increases the generation of ROS in the arterial wall^13^. As previously discussed, oxidized-LDL (ox-LDL) and ROS are the prominent initiators involved in atherosclerosis development.

Aspirin and statin are the most widely used drugs for atherosclerosis prevention and therapy. The pharmacological mechanism of aspirin is to prevent the formation of cyclooxygenase (COX)-dependent vasoconstrictors from thrombosis. Although aspirin shows antithrombotic potency, it also leads to adverse side effects, including gastrointestinal or renal toxicity, hypertension, and extracranial and intracranial hemorrhages^14,15^. In addition, the European Atherosclerosis Society consensus in 2015 regarding a serious side effect of statins indicated that statin-associated muscle symptoms (SAMS) with significantly increased serum creatine kinase (CK) may cause a high risk of future CVD^16,17^. Therefore, there is a need to identify advanced therapeutic approaches to inhibit atherosclerosis.

Kefir originates in the Caucasian mountains; it has been used for centuries and is traditionally produced by the symbiotic fermentation of milk by various species of *Lactobacillales* and yeasts contained within an exopolysaccharide, protein and biomatrix complex referred to as a kefir grain^18,19^. Kefir products have been shown to exhibit broad health benefits not only in basic research but also clinical treatments, including hyperlipidemia prevention, gastrointestinal disease attenuation, allergy and asthma suppression, *Helicobacter pylori* therapeutic improvement, anti-tumor progression applications, and enhancements in bone mineral density of osteoporotic patients^20–27^. These findings indicated the biological activities of kefir in anti-bacterial, antioxidant, anti-tumor and immunomodulating effects. Moreover, the kefir-derived exopolysaccharide kefiran has been demonstrated to reduce systemic cholesterol and blood pressure in spontaneously hypertensive stroke-prone (SHRSP) rats^28,29^. Furthermore, ovalbumin-induced lung inflammation was inhibited by kefiran treatment in a murine model of asthma^30^. The reduced number of macrophages in Peyer’s patches and subsequent mobilization of macrophages relocated to the lamina propria indicate that the oral intake of kefiran may change the balance of macrophages in a mouse model^31^. Our previous study also demonstrated that oral administration of kefir peptides prevents high-fructose corn syrup-induced nonalcoholic fatty liver disease in a murine model via the modulation of inflammation and the JAK2 signaling pathway^32^. These data support the hypothesis that kefir peptides may promote lipid metabolism and anti-inflammatory effects.

The ApoE-deficient gene knockout (*ApoE*^*−/−*^) mouse is the most widely used because of its property of spontaneously developing atherosclerotic lesions by feeding on a regular chow diet^33,34^. An high-fat diet (HFD) was used to accelerate the progression of atherosclerosis. Kefir peptides showed a positive effect on the control of lipid metabolism in HFD-induced obese rats. Therefore, we hypothesize that atherosclerotic lipid deposits may be suppressed in kefir peptides gavage treatment and that kefir peptides may prevent the onset and/or development of atherosclerosis. The objective of this study was to determine the role of kefir peptides in anti-atherosclerosis through oral administration in an HFD-induced *ApoE*^*−/−*^ mouse model.

## Results

### Kefir peptides improve body weight change and systemic lipid profiles in HFD-induced atherosclerosis in *ApoE*^*−/−*^ mouse model

**Figure 1:**
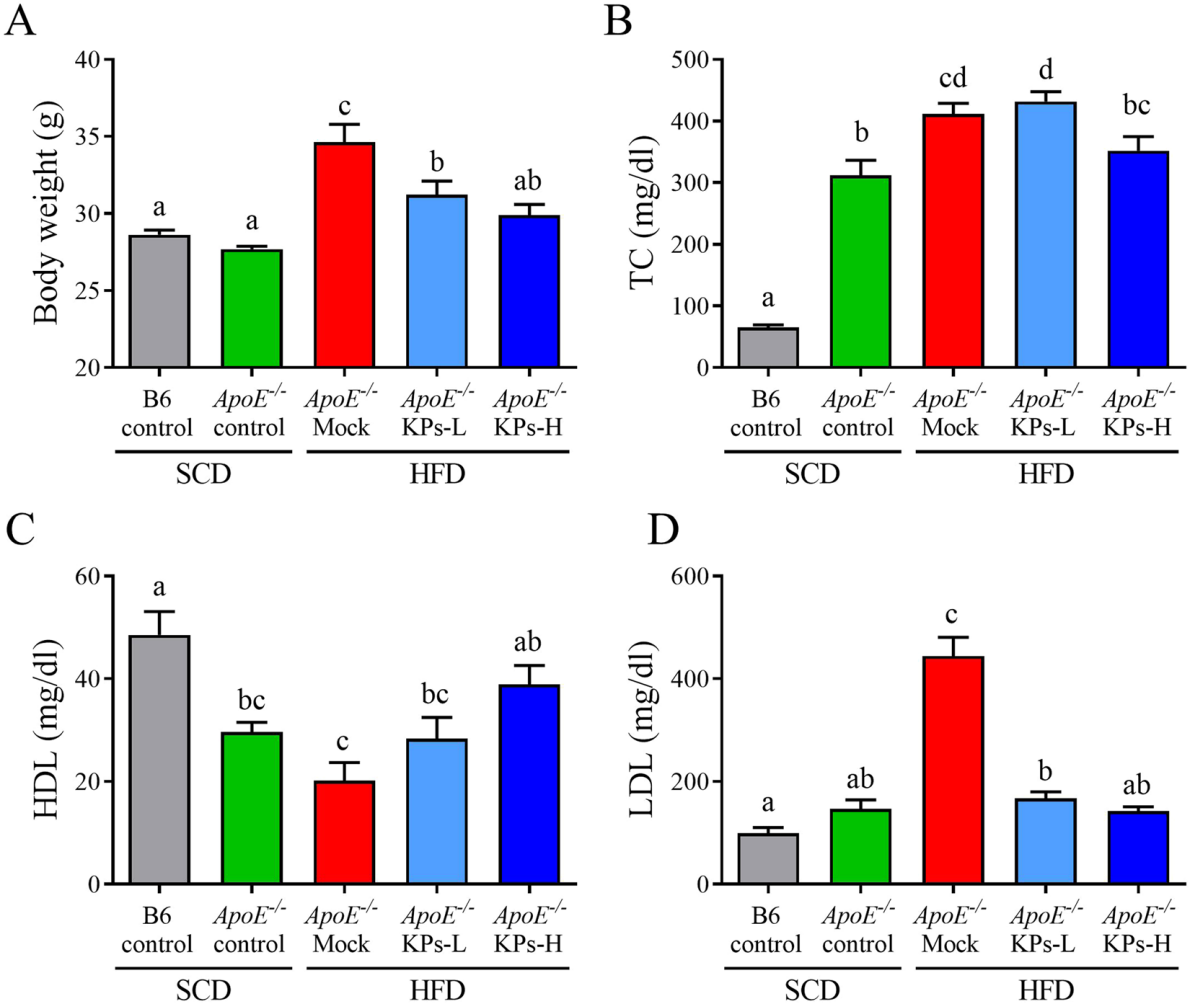
Effects of kefir peptides on body weight change and systemic lipid profiles in HFD-induced atherosclerotic *ApoE*^*−/−*^ mice. (**A**) Weight of mice at 20 weeks of age. Concentrations of (**B**) blood total cholesterol (TC), (**C**) high-density lipoprotein (HDL), and (**D**) low-density lipoprotein (LDL) in different treated mice groups were detected. Data are displayed as the mean ± SEM (n = 8). The statistical analysis was performed according to Duncan’s multiple-range method. The labels at the top of columns without the same letters indicate significant differences between groups (P < 0.05).

The similar initial body weights of the *ApoE*^*−/−*^ mice were randomly divided into all experimental groups (Supplementary Table 1). Food consumption was not significantly different between HFD/Mock and HFD/KPs groups (Supplementary Table 2). After the 12-week treatment, the HFD/*ApoE*^*−/−*^ mock group displayed a 25% increment in body weight when compared with the SCD/*ApoE*^*−/−*^ control group (P < 0.05). Interestingly, kefir peptides (KPs) intake groups exhibited a dose-dependent reduction of body weight, 9.8% lower in low-dose (100 mg/kg) kefir peptides group (KPs-L) and 14.6% lower in high-dose (400 mg/kg) kefir peptides group (KPs-H) group, when compared with the HFD/*ApoE*^*−/−*^ mock group (Fig. 1A). Serum total cholesterol (TC) showed a 3-fold increment in SCD/*ApoE*^*−/−*^ control group when compared with SCD/B6 control groups, while HFD/*ApoE*^*−/−*^ mock group showed 32% higher TC level than SCD/*ApoE*^*−/−*^ control groups (Fig. 1B). Although serum TC showed no significant change in both the HFD/KPs-L and HFD/KPs-H groups when compared with the HFD/*ApoE*^*−/−*^ mock group (Fig. 1B), the concentration of serum high-density lipoprotein (HDL) and low-density lipoprotein (LDL) showed a significantly improve in both dosages of KPs treatment groups (Fig. 1C,D). Serum HDL showed a 40% reduction in SCD/*ApoE*^*−/−*^ control group when compared with SCD/B6 control groups, and further lower serum HDL level was detected in HFD/*ApoE*^*−/−*^ mock group (P < 0.05). Administration of KPs exhibited a dose-dependent upregulation of serum HDL, 40% higher in KPs-L group and 92% higher in KPs-H group, when compared with the HFD/*ApoE*^*−/−*^ mock group (Fig. 1C). In addition, serum LDL showed no significant difference between SCD/*ApoE*^*−/−*^ and SCD/B6 control groups, while HFD/*ApoE*^*−/−*^ mock group showed a 2.2-fold higher serum LDL level than SCD/*ApoE*^*−/−*^ control groups. Administration of KPs showed a 60% reduction of serum LDL level in both of the KPs-L and KPs-H groups when compared with the HFD/*ApoE*^*−/−*^ mock group (P < 0.05; Fig. 1D). Administration of high-dose KPs had a better inhibitory effect on body weight increment and better effects on modulation the serum HDL and LDL level when compared with the HFD/*ApoE*^*−/−*^ mock group (Fig. 1).

### Kefir peptides inhibit atherosclerotic formation in HFD-induced atherosclerotic *ApoE*^*−/−*^ mice

**Figure 2:**
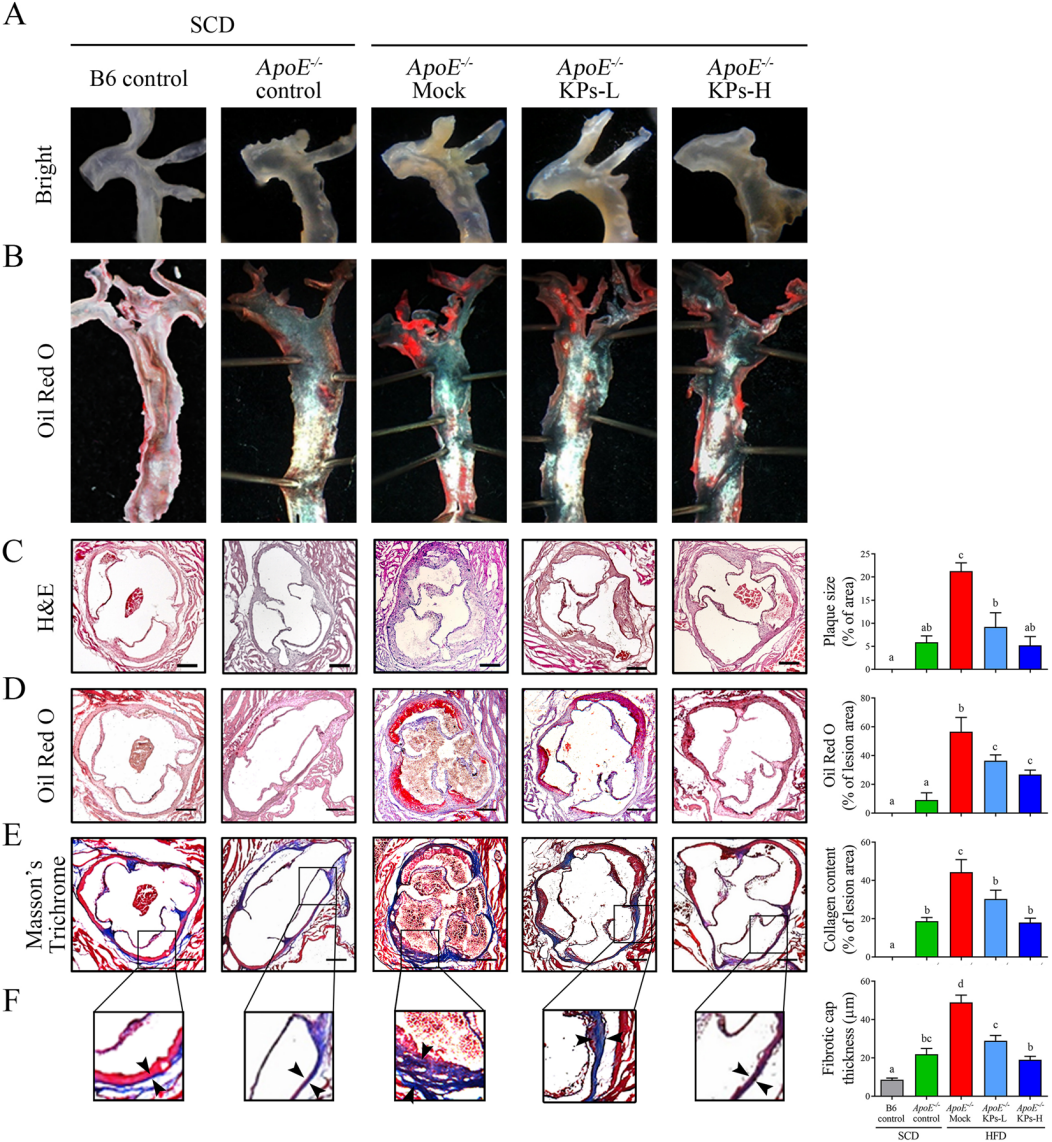
Kefir peptides inhibit atherosclerosis formation in HFD-induced atherosclerotic *ApoE*^*−/−*^ mice. The accumulations of lipid in the whole aortas of *ApoE*^*−/−*^ mice were observed by (**A**) bright view and (**B**) Oil red-O staining. (**C**) Representative images of H&E-stained atherosclerotic lesions and quantification of plaque size demonstrated as the percentage of lesion area within aortic root area. (**D**) Representative images of Oil red O–stained aortic root sections and quantification of lipid deposition within lesion area. (**E**) Representative images of Masson’s Trichrome-stained aortic root sections focused on collagen content and (**F**) plaque fibrotic caps, with quantification of collagen deposition and fibrotic cap thickness, respectively. Scale bar: 200 μm. The statistical analysis was performed according to Duncan’s multiple-range method. The labels at the top of dot plots without the same letters indicate significant differences between groups (P < 0.05).

Atherosclerotic plaques are composed of a lipid-rich core covered with a thin fibrous cap, containing sparse smooth muscle cells and extensive macrophages accumulation^35^. To visualize lipid deposition in the atherosclerotic plaques, aortas were stained and observed with Oil red O staining. As shown in Fig. 2A,B, both HFD/*ApoE*^*−/−*^ KPs groups exhibited significantly less lipid deposition in aortic roots but no effect on thoracic portion of aortas compared with HFD/*ApoE*^*−/−*^ mock group. The atherosclerotic lesion size was examined by the percentage of area of atherosclerotic plaque compared to the whole cross-sectional aortic sinus area stained with H&E (Fig. 2C). The atherosclerotic lesions showed a 2.6-fold increment in HFD/*ApoE*^*−/−*^ mock group compared with the SCD/*ApoE*^*−/−*^ control group. Administration of KPs showed a significant less lesion area in a dosage manner, 56% lower in KPs-L group and 75% lower in KPs-H group, when compared with the HFD/*ApoE*^*−/−*^ mock group (Fig. 2C).

Moreover, Oil red-O staining showed that small amounts of red-stained lipid deposition could be detected in the aortic root in the SCD/*ApoE*^*−/−*^ control group, while a 5.2-fold increment could be detected in HFD/*ApoE*^*−/−*^ mock group compared with SCD/*ApoE*^*−/−*^ control group. Administration of KPs inhibited lipid deposition in the aortic roots in a dose-dependent effect, 35% lower in KPs-L group and 52% lower in KPs-H group, when compared with the HFD/*ApoE*^*−/−*^ mock group (P < 0.05; Fig. 2D). Aortic walls in the HFD/*ApoE*^*−/−*^ mock group exhibited a 1.3-fold increment of collagen and smooth muscle fibers production and deposition (Fig. 2E) and the a 1.2-fold increment on plaque fibrotic caps thicknesses (Fig. 2F) compared with SCD/*ApoE*^*−/−*^ control group. As anticipated, the deposition of collagen content and the fibrous caps of plaques in the KPs administration groups were thinner and contained less collagen in a dose-dependent manner, 40% thinner/31% lower in KPs-L group and 61% thinner/59% lower in KPs-H group, respectively, when compared with the HFD/*ApoE*^*−/−*^ mock group (Fig. 2E,F). Histopathological results indicated that administration of high-dose KPs had a better effect on reduction of lesion area, lipid deposition, and plaque fibrotic caps thicknesses in the atherosclerotic plaques in aortic roots, which showed no discernible difference in the atherogenic levels to the SCD/*ApoE*^*−/−*^ control group (Fig. 2).

### Kefir peptides protect against endothelial dysfunction in HFD-induced atherosclerotic *ApoE*^*−/−*^ mice

**Figure 3:**
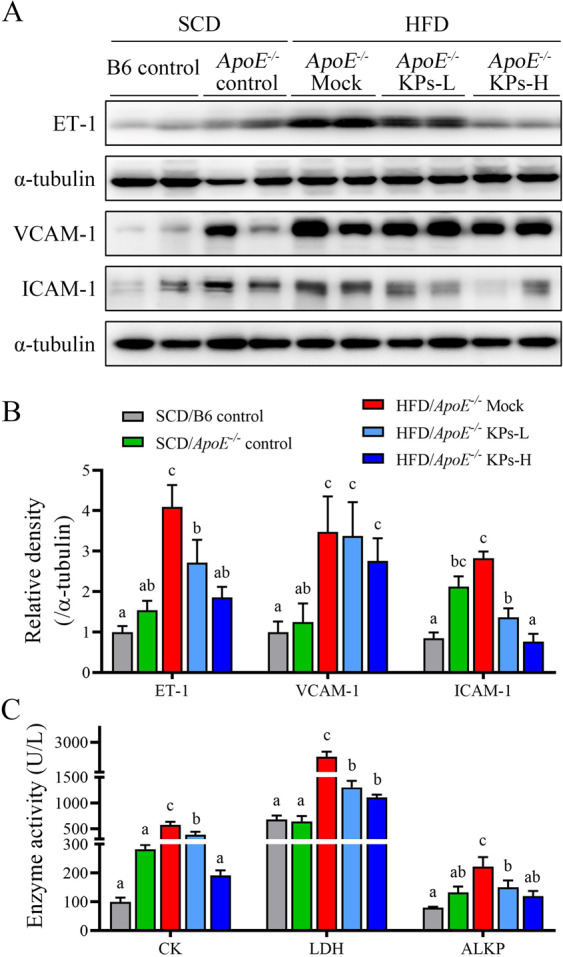
Kefir peptides protect against endothelial dysfunction in HFD-induced atherosclerotic *ApoE*^*−/−*^ mice. (**A**) Western blot analysis was performed to detect endothelial markers, ET-1, VCAM-1, and ICAM-1, protein expressions in the aortic tissue of mice. (**B**) The histogram shows the quantitative densitometry data of the Western blot analysis determined by ImageJ system. Total α-tubulin was used as an internal quantitative control. (**C**) Serum markers for myocardial injury, creatine kinase (CK), lactate dehydrogenase (LDH), and alkaline phosphatase (ALKP), were determined. Data are displayed as the mean ± SEM (n = 8). The statistical analysis was performed according to Duncan’s multiple-range method. The labels at the top of columns without the same letters indicate significant differences between groups (P < 0.05).

Studies demonstrated that endothelin-1 (ET-1) and adhesion molecules (VCAM-1 and ICAM-1) derived from arterial cells is involved in the atherosclerotic development^36^. These markers significant overexpressed in the aortic tissue in HFD/*ApoE*^*−/−*^ mock group compared with SCD/*ApoE*^*−/−*^ control group (P < 0.05; Fig. 3A,B). Oral administration of KPs significantly inhibited the aortic ET-1 and ICAM-1expression levels compared with the HFD/*ApoE*^*−/−*^ mock group (P < 0.05), but no significant change in VCAM-1 level among the HFD/*ApoE*^*−/−*^ mock and HFD/*ApoE*^*−/−*^ KPs groups (Fig. 3A,B). Serum cardiac markers of creatine kinase (CK), lactate dehydrogenase (LDH) and alkaline phosphatase (ALKP) are additional markers for myocardial injury detection^37,38^. These serum markers showed a significant increment (2.9-fold increment in CK level, 1-fold increment in LDH level and 68% increment in ALKP level) in the HFD/*ApoE*^*−/−*^ mock group compared with the SCD/*ApoE*^*−/−*^ control group (P < 0.05; Fig. 3C). However, these serum markers dose-dependently decreased in KPs administration groups (CK level: 48% lower in KPs-L and 56% lower in KPs-H; LDH level: 34% lower in KPs-L and 67% lower in KPs-H; ALKP level: 33% lower in KPs-L and 47% lower in KPs-H) compared with HFD/*ApoE*^*−/−*^ mock group (Fig. 3C). Moreover, molecular markers for atherosclerotic development, ET-1 and ICAM-1, and serum makers for myocardial injury showed a promising improvement of high-dose KPs group when compared with the HFD/*ApoE*^*−/−*^ mock group (Fig. 3).

### Kefir peptides decrease oxidative stress in HFD-induced atherosclerotic *ApoE*^*−/−*^ mice

**Figure 4:**
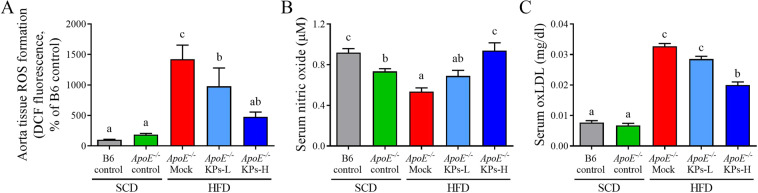
Kefir peptides decrease oxidative stress in HFD-induced atherosclerotic *ApoE*^*−/−*^mice. (**A**) DCF assay to evaluate ROS levels in aortic tissues. Concentrations of (**B**) nitric oxide (NO) and (**C**) ox-LDL in serum were detected. Data are displayed as the mean ± SEM (n = 8). The statistical analysis was performed according to Duncan’s multiple-range method. The labels at the top of columns without the same letters indicate significant differences between groups (P < 0.05).

The HFD/*ApoE*^*−/−*^ mock group showed a 27% reduction of reduction of NO production resulted in a 6.6-fold increment of ROS activity, as assessed by the increase of DCF fluorescence; whilst, their downstream ox-LDL level also showed a 3.8-fold increment when compared with the SCD/*ApoE*^*−/−*^ control group (Fig. 4A–C). Administration of KPs showed a dose-dependent improvement in elevating NO production, decreasing ROS activity and ox-LDL level when compared with HFD/*ApoE*^*−/−*^ mock group (NO level: 28% higher in KPs-L and 75% higher in KPs-H; ROS level: 31% lower in KPs-L and 66% lower in KPs-H; ox-LDL level: 13% lower in KPs-L and 39% lower in KPs-H) (Fig. 4A–C). Administration of high-dose KPs had better inhibitory effects on ROS activities and serum oxLDL level when compared with the HFD/*ApoE*^*−/−*^ mock group (Fig. 4).

### Kefir peptides reduce plaque macrophage accumulation and modulate inflammatory response in HFD-induced atherosclerotic *ApoE*^*−/−*^ mice

**Figure 5:**
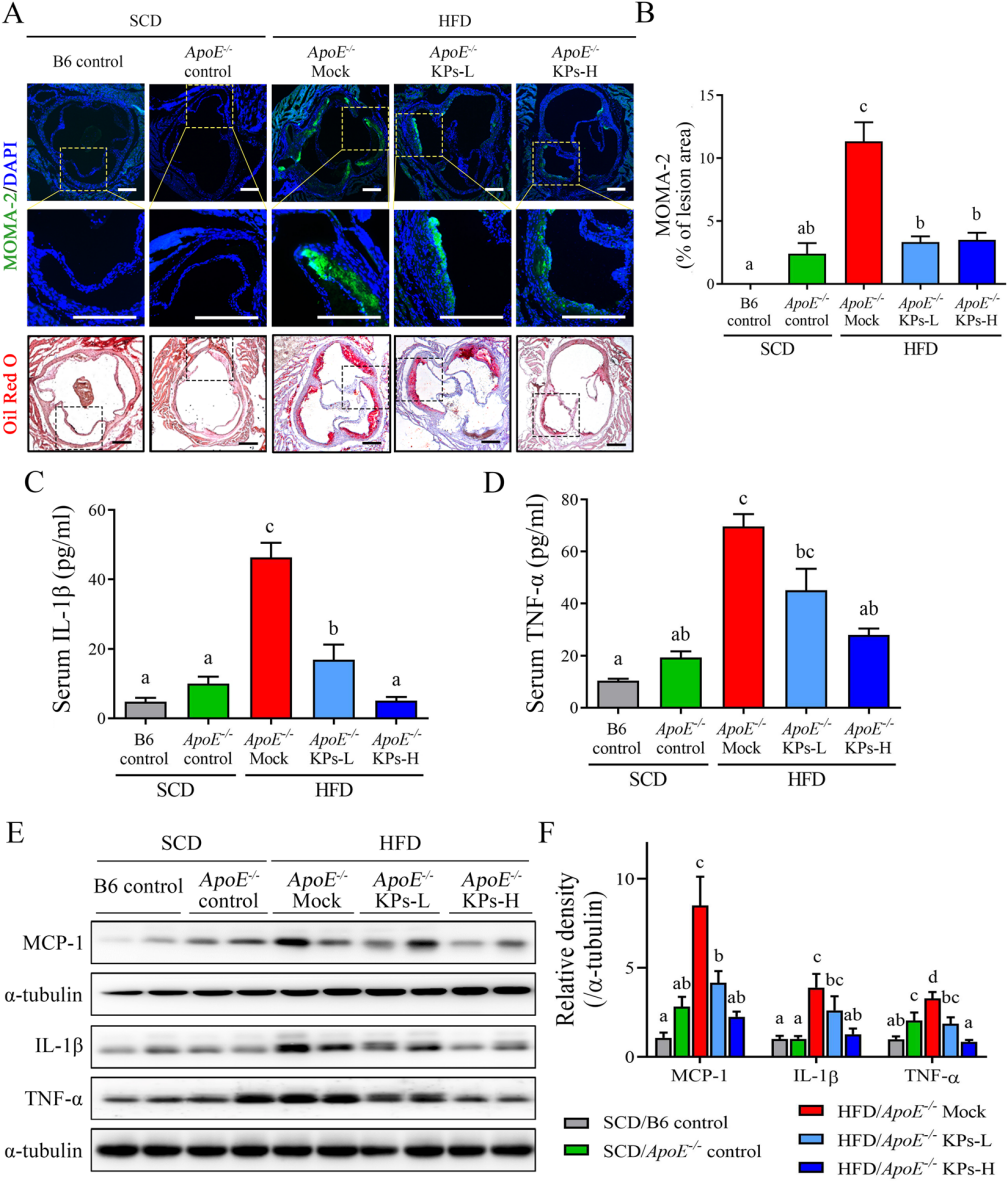
Kefir peptides attenuate monocyte/macrophage infiltration and inflammatory cytokine expressions in HFD-induced atherosclerotic *ApoE*^*−/−*^ mice. (**A**) Representative images showing the lesion content of MOMA-2^+^ macrophage (upper panel) or lipid deposition (lower panel) in each group. Sections of aortic roots were stained with MOMA-2 antibody and Oil red O to visualize macrophage distribution within lesion area. The rectangles in the (**A**) upper and lower panels indicate the same area we chose, which are magnified in the middle panel. Macrophages were stained with MOMA-2 (green); Nuclear was stained with DAPI (blue). Scale bar: 200 μm. (**B**) The histogram shows the quantification data of macrophage infiltration within lesion area. Proinflammatory cytokine levels of IL-1β and TNF-α in serum (**C** and **D**) and in aortic tissues (**E**) were determined by ELISA and Western blot analysis, respectively. Aortic MCP-1 protein expression was also evaluated by Western blot analysis (**E**). (**F**) The histogram shows the quantitative densitometry data of the Western blot analysis determined by ImageJ system. Data are displayed as the mean ± SEM (n = 8). The statistical analysis was performed according to Duncan’s multiple-range method. The labels at the top of columns without the same letters indicate significant differences between groups (P < 0.05).

We further examined whether KPs inhibited plaque formation through modulating inflammatory response and attenuating macrophage infiltration and accumulation. In the present study, an intracellular macrophage marker, MOMA-2, was used to verify macrophage accumulation in atherosclerotic plaques. The HFD/*ApoE*^*−/−*^ mock group showed a 12-fold increment in the content of macrophage accumulation in the lipid-rich site of atherosclerotic plaque, thereby contributing to increased plaque vulnerability when compared with the SCD/*ApoE*^*−/−*^ control group. Administration of KPs showed a dose-dependent reduction (76% lower in KPs-L and 86% lower in KPs-H) in the macrophage accumulation compared to HFD/*ApoE*^*−/−*^ mock group (P < 0.05; Fig. 5A,B).

Several inflammatory cytokines, such as interleukin-1β (IL-1β) and tumor necrosis factor-α (TNF-α), involved in the pro-inflammatory signaling in the atherosclerosis progression^39^. In this study, both IL-1β and TNF-α levels in serum and aortic tissue were detected. In serum, the IL-1β and TNF-α levels were increased by 3.6-fold and 2.6-fold, respectively, in the HFD/*ApoE*^*−/−*^ mock group compared to the SCD/*ApoE*^*−/−*^ control group (Fig. 5C,D). Furthermore, the IL-1β and TNF-α levels in aortic tissue were analyzed via western blot analysis (Fig. 5E). The aortic protein expression levels of IL-1β and TNF-α were increased by 4-fold and 1.5-fold, respectively, in the HFD/*ApoE*^*−/−*^ mock group compared to the SCD/*ApoE*^*−/−*^ control group (Fig. 5F). Administration of KPs showed a dose-dependent reduction of IL-1β and TNF-α levels in serum and aortic tissue compared to HFD/*ApoE*^*−/−*^ mock group (Fig. 5C–F). In addition, monocyte chemoattractant protein-1 (MCP-1), which can recruit monocyte/macrophage to the site of vascular inflammation, was significantly decreased in the both dosages of KPs administration groups when compared with the HFD/*ApoE*^*−/−*^ mock group (Fig. 5E,F). The amazing results indicated that the high-dose of kefir peptides (HFD/*ApoE*^*−/−*^ KPs-H) had a greater effect on inhibiting macrophage accumulation and modulating inflammatory response in the aorta, which showed no discernible difference in the levels of inflammation to the SCD/*ApoE*^*−/−*^ control group (Fig. 5).

### Kefir peptides suppress endothelial cell activation and THP-1 monocytes adhesion and migration under ox-LDL-conditioned cell cultures

**Figure 6:**
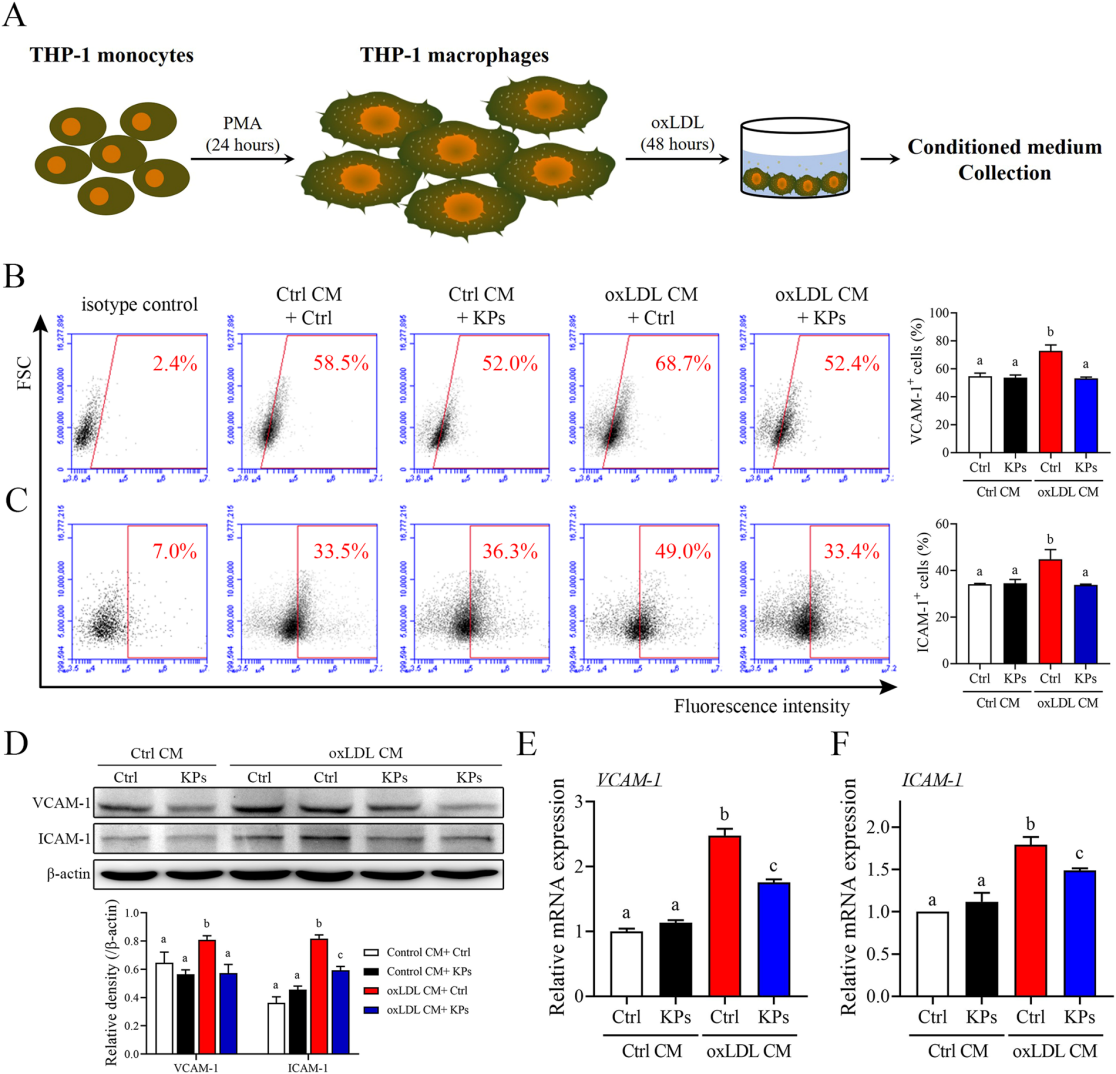
Effects of kefir peptides on endothelial adhesion molecules. (**A**) Flow chart of the conditioned medium (oxLDL CM) collection from ox-LDL-stimulated THP-1 macrophages. Conditioned medium from unstimulated THP-1 macrophages were used as a control (Ctrl CM). (**B**, **C**) Expression of adhesion molecules, VCAM-1 and ICAM-1, in HUVECs after 6 h incubation in oxLDL CM or Ctrl CM with or without kefir peptides (KPs, 100 μg/ml) were determined by flow cytometry. The histograms on the (**B** and **C**) right show the quantification data of three independent HUVECs samples of VCAM-1 or ICAM-1-antibody staining, respectively. (**D**) Western blot analysis of VCAM-1 and ICAM-1 protein expressions. The histogram on the lower panel shows the quantitative densitometry data of the Western blot analysis determined by ImageJ. Quantitative mRNA expressions of VCAM-1 (**E**) and ICAM-1 (**F**) were performed by real-time RT-PCR analysis. Values were normalized to the β-actin gene and are expressed relative to the control (Ctrl) group. The statistical analysis was performed according to Duncan’s multiple-range method. The labels at the top of columns without the same letters indicate significant differences between groups (P < 0.05).

Overexpression and accumulation of ox-LDL in the arterial wall and subsequent monocyte trafficking across the vessel wall to differentiate into macrophages are critical steps during the atherosclerotic plaque development^40,41^. To mimic these steps, phorbol 12-myristate 13-acetate (PMA)-induced THP-1 macrophages were used and then treated with ox-LDL for 48 h and conditioned medium (CM) was collected (Fig. 6A). These ox-LDL CM were used to study the potential inhibitory effects of KPs on the early atherosclerotic processes. As anticipated, ox-LDL CM evoke endothelial activation as shown by upregulation of adhesion molecules mRNA and protein levels in HUVECs (Fig. 6). Accordingly, addition of KPs to the ox-LDL CM showed a significant inhibitory effect on upregulation of endothelial adhesion molecules VCAM-1 and ICAM-1 mRNA expressions (29% and 40% lower, respectively) as well as protein levels (flow cytometry: 40% and 25% lower; western blot: 29% and 27% lower, respectively) in HUVECs after 6 h incubation (Fig. 6B–F). Results showed that KPs had a potential inhibitory effect on oxLDL-stimulated endothelial activation.

**Figure 7:**
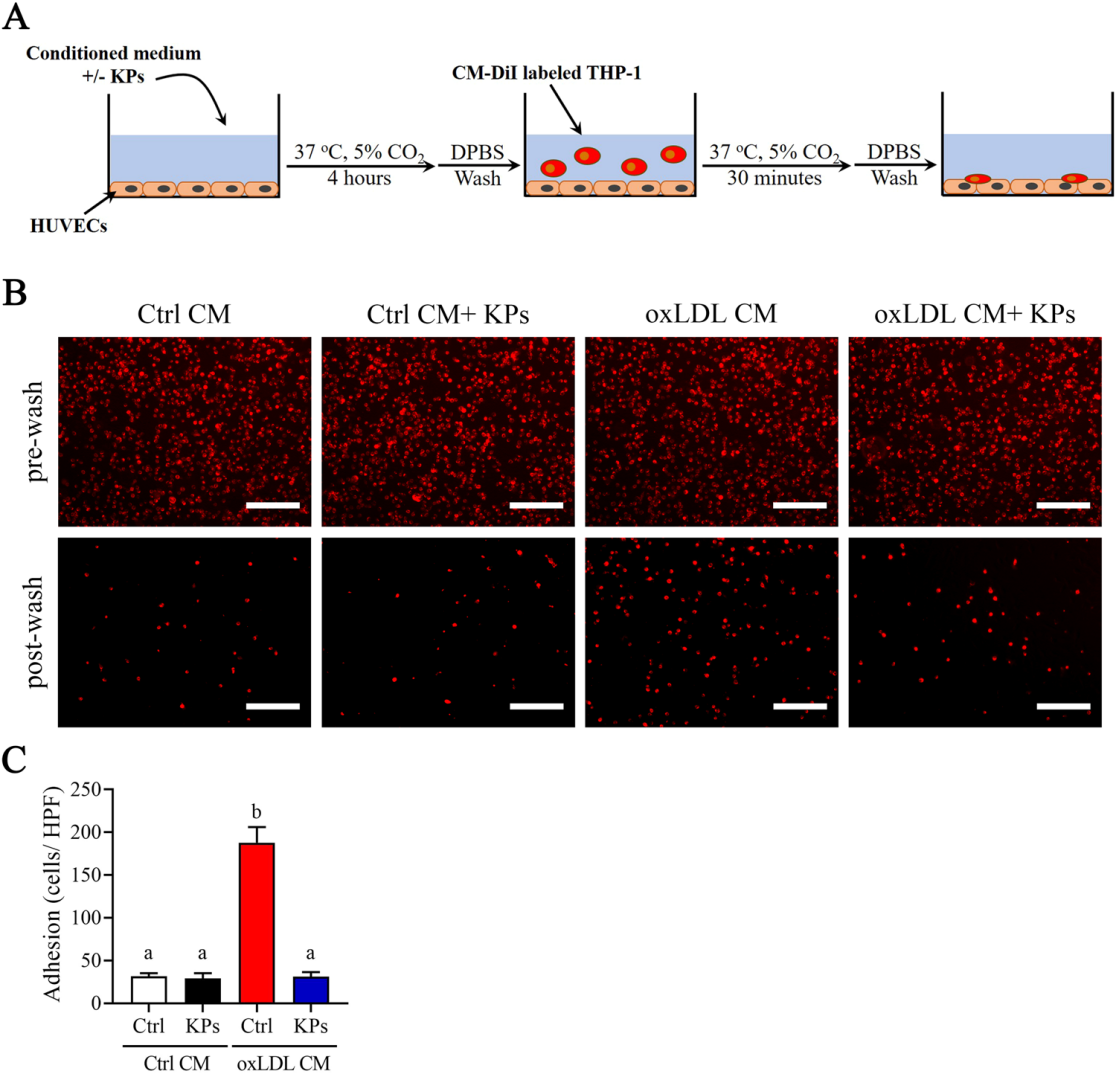
Effects of kefir peptides on adhesion of THP-1 monocytes to endothelial cells. (**A**) Flow chart of the experimental setting. (**B**) Representative fluorescent images showing CM-DiI-labeled THP-1 monocytes on HUVECs monolayer after 4 h incubation in oxLDL CM or Ctrl CM with or without kefir peptides (KPs, 100 μg/ml) followed by addition of red fluorescent CM-DiI labeled THP-1 monocytes. Pictures before (upper panel) and after (lower panel) DPBS wash are taken. (**C**) The histogram shows the quantification data of three independent adherent cells per high power field (HPF) after washing determined by ImageJ. Ctrl CM: conditioned medium from unstimulated THP-1 macrophages as a control. Scale bar: 200 μm. The statistical analysis was performed according to Duncan’s multiple-range method. The labels at the top of columns without the same letters indicate significant differences between groups (P < 0.05).

**Figure 8:**
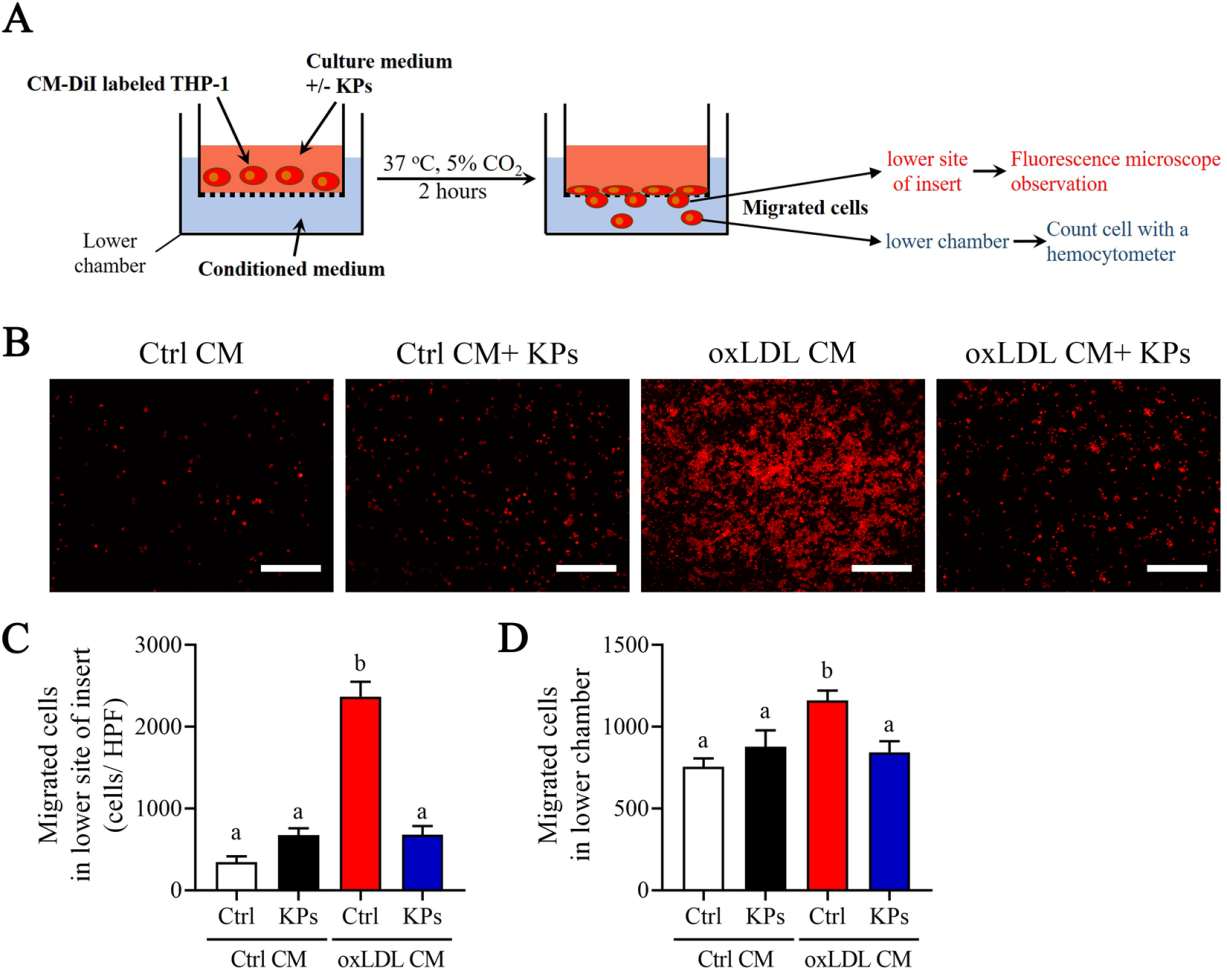
Effects of kefir peptides on migration of THP-1 monocytes. (**A**) Flow chart of the experimental setting. (**B**) Representative fluorescent images showing CM-DiI-labeled THP-1 monocytes which migrated across the transwell insert membrane to the lower site after 2 h incubation in oxLDL CM or Ctrl CM with or without kefir peptides (KPs, 100 μg/ml). (**C**) The histogram shows the quantification data of three independent migrated cells on the lower site of transwell insert per high power field determined by ImageJ. Scale bar: 200 μm. (**D**) The histogram shows the number of three independent migrated cells pass through the transwell insert which suspend in the lower chamber were counted using hemocytometer. The statistical analysis was performed according to Duncan’s multiple-range method. The labels at the top of columns without the same letters indicate significant differences between groups (P < 0.05).

We next investigated the inhibitory effects of KPs on the adhesion and following migration ability of THP-1 monocytes (Fig. 7A). Interestingly, THP-1 monocyte strongly adheres to a confluent monolayer of ox-LDL CM pre-incubated HUVECs under static conditions. However, addition of KPs to the ox-LDL CM pre-incubated HUVECs showed an 83% inhibitory efficacy on THP-1 monocytes adhesion (Fig. 7A,B). Furthermore, we investigated THP-1 monocytes migration ability using the transwell system (Fig. 8). Results showed that ox-LDL CM attract lots of THP-1 monocytes to the lower sites of insert and lower chamber, while a significant inhibitory effect was detected (71% and 27% reduction of monocyte in lower site of insert and lower chamber, respectively) under KPs treatment (Fig. 8). Taken together, KPs addition strongly inhibit the monocyte adhesion to endothelial and subsequent migration into the subendothelial region (Figs. 7 and 8).

## Discussion

**Figure 9:**
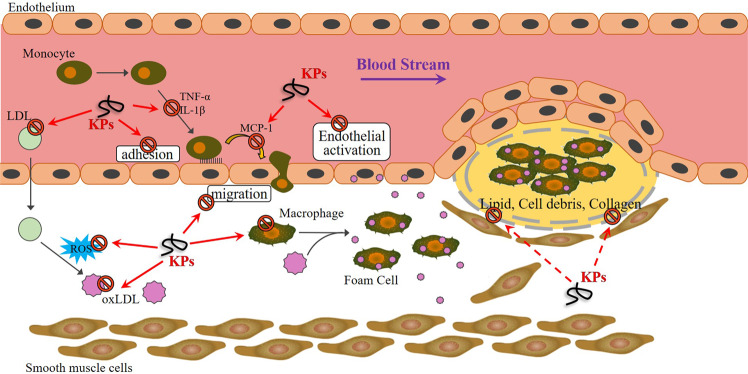
Schematic action of kefir peptides on suppression of HFD-induced atherosclerotic progression. The potential action of kefir peptides on anti-atherosclerosis progression through protection against endothelium dysfunction, inhibition of monocyte adhesion/migration, inflammatory response, ox-LDL generation, and oxidative stress, followed by attenuation of aortic lipid deposition, macrophage accumulation in plaques and heart fibrosis.

Despite the benefits of aspirin and statin, which are well-established in CVD prevention and therapy, the possibility of aftereffects must be considered^14,16^. In this study, we first demonstrated anti-atherosclerotic progression by kefir peptides consumption in *ApoE* knockout mice. A substantial increase in aortic lipid deposition, oxidative stress, plaque macrophage accumulation, systemic inflammatory response and aortic fibrosis were induced by HFD-induced atherosclerosis. The beneficial of HFD-induced atherosclerotic mouse model than spontaneously developing atherosclerotic lesions by SCD-fed *ApoE*^*−/−*^ mouse model is that HFD can accelerate the progression of atherosclerosis and also elevate AST and ALT levels (Supplementary Fig. 1A,B) to increase the risk of developing CVD. Treatment with kefir peptides completely indicated an anti-atherogenic effect in a dose-dependent manner. Our investigation suggested fruitful effects of kefir peptides to support novel therapy and prevention approaches for anti-atherosclerotic effects. The proposed mechanism of kefir peptides in atherosclerosis treatment are shown in Fig. 9.

Considering human atherosclerosis development takes from months to years or even decades with individual variations, thus two ideal strains of mice, *ApoE*^*−/−*^ and LDL receptor deficient (*Ldlr*^*−/−*^) mice, to study the atherosclerosis are established, which are susceptible to develop atherosclerotic lesion formation during high-fat or high-cholesterol diet and several features of the disease mimic to humans^42^. Although both strains develop features of type 2 diabetes and promote atherosclerosis development, *Ldlr*^*−/−*^ mice are more prone to develop diabetic phenotype including increased body weight, subcutaneous fat accumulation, high blood glucose, and developed an insulin resistance compared to the *ApoE*^*−/−*^ mice and the control wild-type mice during HFD challenge^43^. On a standard chow diet, *ApoE*^*−/−*^ mice showed higher total cholesterol level in plasma compared with *Ldlr*^*−/−*^ and the control wild-type mice, similar result detected in Fig. 1B, and eventually develop atherosclerotic lesions a few months after birth^44^. Due to the imbalance of the cholesterol deposition in the macrophage and tissue which trigger side effects related to inflammation and extracellular matrix degradation, such as Alzheimer’s, steatohepatitis, and respiratory diseases^45^. Therefore, the choice of the diet is very important. A high-cholesterol diet but not high-fat diet in given to *ApoE*^*−/−*^ mice with high risk factors for neurodegeneration^46^, Alzheimer disease^47,48^, retinal abnormalities^49^, and chronic obstructive pulmonary disease (COPD)^45^. Taken together, we chose the high-fat diet to induce atherosclerosis in *ApoE*^*−/−*^ mouse model in this study.

Our previous *in vivo* animal study demonstrated that kefir peptides prevent hyperlipidemia in HFD-induced obese rats through the inhibition of the lipogenesis pathway through reduced fatty acid synthase (FAS) enzyme, increased p-ACC protein, and stimulation of the lipid oxidation pathway via augmented expression of p-AMPK, PPAR-α, and CPT1^50^. Moreover, we found that kefir peptides improved non-alcoholic fatty liver diseases through manipulation of the JAK2/STAT3 and JAK2/AMPK signaling pathways in a high fructose-induced fatty liver animal model^32^. These current studies indicated that kefir peptides play an important role in lipid metabolism modulation. The precise detection of lipid deposition is critical for monitoring atherosclerotic progression. Although circulating cholesterol accumulation in HFD-induced atherosclerosis was not suppressed by kefir peptide treatment, the aortic lipid deposition was dramatically abolished by kefir peptide administration, which suggested that the anti-atherogenic effect by kefir peptides did not occur through the regulation of cholesterol metabolism. Another view of this result suggested that HFD-induced atherosclerosis in *ApoE*^*−/−*^ mice may develop serious atherosclerosis, and the hyperlipidemia was not extremely altered by kefir peptides (Fig. 1B).

ET-1, a potent vascular function indicator, is involved in vasoconstriction, free radical formation and proinflammatory response and results in the development of vascular dysfunction and cardiovascular disease^51^. Furthermore, ox-LDL, induced by ET-1 in human endothelial cells, stimulates ROS generation through NADPH oxidase as previously reported^52,53^. Our results demonstrated that ET-1, ox-LDL, and ROS were significantly decreased by kefir peptide treatment in HFD-induced atherosclerosis mice, which suggested that kefir peptides may have an effect on endothelial function protection (Figs. 3 and 4). Not surprisingly, in a previous study, Friques and his colleagues^54^ also found that kefir ameliorates the endothelial function in spontaneously hypertensive rats (SHR) by restoring the ROS/NO imbalance. Our results showed that no significantly change on the blood pressure (BP), including systolic, diastolic, and mean blood pressure after 12 weeks HFD and KPs administration between each group (Supplementary Fig. 1C-E).

Atherosclerosis is a chronic inflammatory disorder of aortic disease. Expressions of both ICAM-1 and VCAM-1 are produced on endothelial cells of atherosclerotic plaque development by several mediators, including ROS and ox-LDL^55^. More importantly, ICAM-1 and VCAM-1, expressed by abnormal endothelium in developing atherosclerotic plaques, are required for the circulating monocyte recruitment to atherosclerotic lesions^56^. Moreover, VCAM-1 has been shown to play a dominant role in the initiation of atherosclerosis^57^. However, evidence has demonstrated that ICAM-1 deficiency substantially protects against atherosclerosis lesion formation in *ApoE*^*−/−*^ mice^58,59^, indicating a controversial issue, which is the dominate mediator between ICAM-1 and VCAM-1 in modulating atherosclerosis progression. Our results indicated there was no significant difference in VCAM-1 was observed among the HFD/*ApoE*^*−/−*^ mock, HFD/*ApoE*^*−/−*^ KPs-L, and HFD/*ApoE*^*−/−*^ KPs-H groups; however, the expression of ICAM-1 protein was substantially suppressed in both the HFD/*ApoE*^*−/−*^ KPs-L and HFD/*ApoE*^*−/−*^ KPs-H groups compared to the HFD/*ApoE*^*−/−*^ mock group (Fig. 3). Consistently, ICAM-1 inhibition markedly attenuates macrophage homing to atherosclerotic plaques in ApoE-deficient mice^60^, which suggests ICAM-1 may play a leading role in atherosclerosis procession.

Atherosclerotic lesion development is an inflammatory process accompanied by the recruitment and activation of macrophages, which trigger downstream cascade activation and enhance inflammatory cytokine secretion. Abundant evidence indicates that macrophage-mediated inflammation comprises a central role in atherosclerotic development and may trigger acute thrombotic vascular disease, stroke, myocardial infarction, and sudden cardiac death^61,62^. Our study identified macrophages in the blood stream and homing to atherosclerotic lesions through MCP-1 chemoattractant, which is secreted by aortic endothelial cells. Suppressing the accumulation of lesion macrophages by kefir peptide consumption effectively decreased the inflammatory cytokine IL-1β and TNF-α production (Fig. 5). The anti-inflammatory properties of kefir products have been demonstrated in a mouse model and humans^63^. Our results further suggest that kefir peptides may be absorbed into the blood and influence atherosclerotic development through its immune modulation ability.

## Conclusion

In summary, our results indicated that atherosclerotic lesion development in HFD-induced atherosclerotic *ApoE*^*−/−*^ mice was improved by oral administration of kefir peptides. We identified reduced aortic lipid deposition, oxidative stress, macrophage accumulation in plaques, systemic IL-1β and TNF-α levels, and aortic root fibrosis and enhanced endothelial function following kefir peptide intake compared with the SCD/*ApoE*^*−/−*^ control group. Furthermore, the *in vitro* cell studies also demonstrated that kefir peptides suppress endothelial cell activation and THP-1 monocytes adhesion and migration under ox-LDL-conditioned cell cultures. These results suggested that kefir peptides play a role in anti-atherosclerosis potentially by modulating the immune cell responses, reducing ROS and ox-LDL productions, and regulating cytokine related pathways. The profitable impacts of kefir peptides provide new perspectives for its use as an anti-atherosclerotic agent in the preventive medicine.

## Methods

### Experimental animals

C57BL/6 and *ApoE*^*−/−*^ mice purchased from the Jackson Laboratory (Bar Harbor, ME, USA) were maintained on a 12-h light-dark cycle at 22 ± 2°C. This study was conducted according to institutional guidelines and was approved by the Institutional Animal Care and Utilization Committee of the National Chung Hsing University (IACUC No. 104-076), Taiwan. All animal procedures were conformed to the guidelines from Directive 2010/63/EU of the European Parliament on the protection of animals used for scientific purposes. The experimental animals were acclimated to the environment and diet for 2 weeks. At the age of 7 weeks, the male mice were fed a standard chow diet (SCD) (28.5% protein, 13.4% fat, and 58.1% carbohydrates; Cat. No. 5001, LabDiet Co., St. Louis, MO, USA) or an atherogenic high-fat diet (HFD) (18.1% protein, 61.6% fat, and 20.3% carbohydrates; Cat. No. 58Y1, LabDiet Co.) for 12 weeks. The male mice were randomly divided into five treatment groups (n = 8): (1) C57BL/6 mice on a standard chow diet (SCD/B6 control); (2) *ApoE*^*−/−*^ mice on a standard chow diet (SCD*/ApoE*^*−/−*^ control); (3) *ApoE*^*−/−*^ mice on an HFD + PBS treatment as a mock control (HFD/*ApoE*^*−/−*^ mock); (4) *ApoE*^*−/−*^ mice on an HFD + 100 mg/kg low-dose kefir peptides powder (HFD/*ApoE*^*−/−*^ KPs-L); and (5) *ApoE*^*−/−*^ mice on an HFD + 400 mg/kg high-dose kefir peptides powder (HFD/*ApoE*^*−/−*^ KPs-H). Kefir peptides were dissolved in phosphate-buffered saline (PBS; pH 7.4) and orally administered daily for 12 weeks. The mice were sacrificed by intra-peritoneal injection of pentobarbital (60 mg/kg) at 19 weeks of age, after 12 weeks of kefir peptides administration. The heart, aorta, blood, and tissues were collected for further examination.

### Kefir peptides preparation

Kefir starter grains (Phermpep Co., Taichung, Taiwan) were inoculated (5%, wt/vol) and propagated in sterilized milk at 20°C for 20 h to activate them. The grains were retrieved through a sieve, reinoculated (10%, wt/vol) into sterilized fresh milk and incubated following the previously described methods^32,50^. The peptide content in the kefir peptides powder (Phermpep Co., Taichung, Taiwan), calculated as the triglycine equivalent in gram per 100 g sample, was 23.1 g/100 g. The compositions and quality controls of kefir peptides powder for the peptides separation and reproducibility are shown in the Supplementary Fig. 2.

### Determination of biochemical markers

Blood samples were obtained from the retro-orbital sinus using a serum separation tube (SST). Serum was collected following centrifugation of the blood at 10,000 × rpm for 10 min. The levels of total cholesterol (TC), creatine kinase (CK), alkaline phosphatase (ALKP), lactic dehydrogenase (LDH), aspartate aminotransferase (AST), and alanine aminotransferase (ALT) were measured using a VetTest^TM^ automatic colorimetrically analyzer (Idexx Laboratories Inc., Westbrook, ME, USA)^50^. The levels of oxidized low-density lipoprotein (ox-LDL), nitric oxide (NO), reactive oxygen/nitrogen species (ROS/RNS), and inflammatory cytokines, including TNF-α and IL-1β, were determined by ELISA kits (Abcam Inc., Cambridge, MA, USA) according to the manufacturer’s protocol.

### Western blot analysis

The thoracic aortas and cell lines were homogenized in 300 μl of an RIPA buffer (Sigma-Aldrich, St. Louis, MO, USA) for protein extraction. The protein (50 μg) was then separated via 10% SDS-polyacrylamide gel electrophoresis (SDS-PAGE) and electrotransferred onto polyvinylidene difluoride (PVDF) membranes. The membranes were incubated in a blocking solution (5% bovine serum albumin) at room temperature for 2 h, followed by incubation with a primary antibody (MCP-1, ICAM-1, VCAM-1, ET-1, TNF-α, IL-1β, α-tubulin or β-actin; Abcam Inc.) overnight at 4 °C. After washing, the membranes were incubated with anti-rabbit or anti-mouse horseradish peroxidase (HRP) conjugated secondary antibody. The membranes were developed using an enhanced chemiluminescence (ECL) western blot detection system (GE Healthcare Biosciences, Pittsburgh, PA, USA) as previously described^50^.

### Histological and immunofluorescent (IF) staining

The C57BL/6 and *ApoE*^*−/−*^ mice were scarified, the thoracic cavity was opened and perfused with PBS via the left ventricle, and then aortic artery was collected. Aortic sinus tissues were fixed in 4% paraformaldehyde overnight, embedded in paraffin, and cut into sections for hematoxylin and eosin (H&E) staining. Atherosclerotic plaques sizes were examined and quantified from four independent sets of H&E-stained section. Oil red O staining was performed to identify the lipid deposition as previously described^32^. Briefly, frozen aortic sinus tissue sections (10–12 µm) and en face aortic samples were stained with Oil red O (Sigma-Aldrich) for 10 min at 37°C, washed and counterstained with hematoxylin for 1 min to determine lipid accumulation. Representative photomicrographs were captured using Olympus IX71 microscope with an AxioCam MRc camera. The quantification of Oil red O-positive staining area was performed in the middle of 4 sections (slides 3–6) out of 9 serial sections of aortic root (Supplementary Fig. 3) using NIH Image software (ImageJ 1.35 d; NIH, Bethesda, MD, USA)^64^. IF staining was performed with primary antibody of rabbit anti-MOMA-2 (1:50; Abcam Inc.) and Alexa Fluor® 488 conjugated donkey anti-rabbit IgG (Abcam Inc.) according to the manufacturer’s protocol. The slides were mounted with DAPI-Fluoromount-GTM (SouthernBiotech, Birmingham, AL, USA) and analyzed by fluorescence microscopy^65^.

Masson’s trichrome staining was performed to identify the collagen fibers contents in the aortic sinus tissue sections as previously described^65^ A ll analyses were followed the protocol and performed by a pathologist who was blinded to the experimental procedure.

### Cell lines

Human monocytic cell line (THP-1) was purchase from Bioresource Collection and Research Center (Hsinchu, Taiwan). Cell lines were maintained in RPMI-1640 media supplemented with 10% heat-inactivated FBS (Life Technologies Co., Camarillo, CA, USA), 1% penicillin/streptomycin and 50 μM β-mercaptoethanol (Sigma-Aldrich) and were incubated at 37oC in a 5% CO_2_ incubator. Human umbilical vein endothelial cell line (HUVECs) was purchased from Lonza Walkersville, Inc. (Walkersville, MD, USA). Cell lines were maintained in the endothelial cell basal medium (EBM-2, Lonza) supplemented with an endothelial cell growth SingleQuot kit (EGM-2, Lonza) and were incubated at 37 °C in a 5% CO_2_ incubator.

### Preparation of conditioned medium

As shown in Fig. 6A, THP-1 cells were differentiated to adherent macrophages by overnight culture in culture medium supplemented with 100 ng/ml phorbol 12-myristate 13-acetate (PMA, Sigma-Aldrich), and then with 35 μg/mL ox-LDL (Life Technologies Co.) for 48 h. After stimulation, supernatant was collected and removed cell debris by centrifugation and passed through 0.2 μm filters. The conditioned medium is referred as an ox-LDL CM and the collected medium from unstimulated THP-1 macrophages is referred as a control CM.

### Adhesion assay

As shown in Fig. 7A, 3×10^4^ HUVECs were seeded on 48 well plate in EBM-2/ EGM-2 medium, to complete confluence. Cells were incubated with ox-LDL CM with or without KPs (100 μg/ml) for 4 h. After stimulation, HUVECs were washed twice with DPSB and 5×10^5^ CM-DiI-labeled THP-1 monocytes were added to each well of a 48 well plate and incubated at 37 °C in a 5% CO_2_ incubator for 30 min. Then, each well was washed three times with DPBS and high-power field (HPF) digital images were captured using Olympus IX71 microscope with an AxioCam MRc camera. Adhered cells per HPF was counted and calculated using ImageJ software (National Institute of Health, USA).

### Migration assay

As shown in Fig. 8A, 1×10^5^ CM-DiI labeled THP-1 monocytes were added to transwell inserts (Millipore, Danvers, MA, USA) with 8 μm pores and incubated in complete medium with or without KPs (100 μg/ml). Assemble transwell inserts in the chamber of 24-wells culture plate with ox-LDL CM or control CM and incubated at 37 °C in a 5% CO_2_ incubator for 2 h. Cells on the top of the transwell insert were removed using a cotton swab and only migrated cells on the lower site membrane of transwell insert or suspend in the lower chamber were analyzed. The transwell insert membrane were carefully cut and mounted with FluoreGuard Mounting Medium (Biosystems, Barcelona, Spain) on a glass slide. The high-power field (HPF) digital images were taken using Zeiss AxioScope A1 microscope with an AxioCam MRc camera. Cells per HPF was counted and calculated using ImageJ software. To determine the migrated cell numbers in lower chamber using the haemacytometer.

### Flow cytometry analysis

Flow cytometry was used to examine the ICAM-1 and VCAM-1 expressions on the endothelial cells surface according to manufacturer’s instructions (Abcam Inc.). Briefly, detached cells were fixed with 10% formalin for 20 min, and then incubated 1 h at room temperature with the following antibodies at appropriate dilutions in 2% Tween-20 with 2% BSA in PBS: ICAM-1 (1 μg/test) and VCAM-1 (1:40 dilutions). The cells were then incubated with an appropriate Alexa Fluor® 488 and Alexa Fluor® 633 dye-conjugated secondary antibody (1:500 dilutions) at room temperature for 30 min. After rinsing the cells twice, fluorescence was detected and analyzed using BD Accuri™ C6 Plus flow cytometry (BD Biosciences, Franklin Lakes, NJ, USA).

### RNA isolation and quantitative real-time RT-PCR

Total RNA was prepared from cell lines using the Presto™ DNA RNA Protein Extraction Kit (Geneaid Biotech Ltd., Taipei, Taiwan). RNAs were reverse transcribed into cDNAs using an MMLV Reverse Transcription kit (Protech, Sparks, NV, USA). Quantitative real-time RT-PCR was performed using qPCRBIO SyGreen Mix and the Rotor-Gene 6000 cycler (Qiagen Inc., Germantown, MD, USA). Relative gene expression was determined by the ∆∆Ct method, where Ct is the threshold cycle. The relative mRNA expression levels were normalized to the mRNA level of the reference β-actin gene. Primer sequences are listed as follows: VCAM-1 forward: 5′-GCAAGTCTACATATCACCCAAG-3′ and VCAM-1 reverse: 5′-TCACAGAGCCACCTTCTT-3′; ICAM-1 forward: 5′-CCGGAAGGTGTATGAACTG-3′ and ICAM-1 reverse: 5′-TCCATGGTGATCTCTCCTC-3′, β-actin forward: 5′-GCGAGAAGATGACCCAGATC-3′ and β-actin reverse: 5′-CCAGTGGTACGGCCAGAGG-3′.

### Statistical analysis

All data are expressed as the mean ± SEM. The statistical analysis was performed according to Duncan’s multiple-range method to detect differences in the parameters among the control and treatment groups using Prism software (Prism 8.0, GraphPad Software, Inc., San Diego, CA, USA) and SPSS (Statistical Product and Service Solutions; IBM., New York, NY, USA). The labels at the top of columns or dot plots without the same letters indicate the significant difference between each group. The threshold for statistical significance was P < 0.05.

### Ethics approval and consent to participate

All animal experiments were performed according to the guidelines and were approved by the Institutional Animal Care and Utilization Committee of National Chung Hsing University, Taiwan (IACUC No. 104–076).

## Supplementary information

Supplementary Information.

## Data Availability

All data and materials are included in the article and its supplementary information files.
