# Supplementary material for: Kefir peptides alleviate high-fat diet-induced atherosclerosis by attenuating macrophage accumulation and oxidative stress in ApoE knockout mice
Source: Sci Rep. 2020 May 29;10:8802. doi: 10.1038/s41598-020-65782-8 (PMC7260220; doi:10.1038/s41598-020-65782-8)
Supplement: Supplementary file 1 — Supplementary Information. [file 41598_2020_65782_MOESM1_ESM.pdf]

Scientific Reports

Supporting Information

Kefir peptides alleviate high-fat diet-induced atherosclerosis by attenuating macrophage accumulation and oxidative stress in *ApoE* knockout mice

Min-Che Tung[1,2,3†], Ying-Wei Lan[1†], Hsin-Han Li[1†], Hsiao-Ling Chen[4], Sheng-Yi Chen[1], Yu-Hsuan Chen[1], Chi-Chien Lin[5], Min-Yu Tu[6,7,8], and Chuan-Mu Chen[1,9]*

[1] Department of Life Sciences, and Ph.D. Program in Translational Medicine, National Chung Hsing University, Taichung 402, Taiwan;

[2] Department of Surgery, Tungs' Taichung Metro Harbor Hospital, Taichung 435, Taiwan;

[3] Graduate Institute of Clinical Medicine, Taipei Medical University, Taipei 110, Taiwan;

[4] Department of Bioresources, Da-Yeh University, Changhwa 515, Taiwan;

[5] Institute of Biomedical Sciences, National Chung Hsing University, Taichung 402, Taiwan;

[6] Aviation Physiology Research Laboratory, Kaohsiung Armed Forces General Hospital Gangshan Branch, Kaohsiung 820, Taiwan

[7] Department of Biomedical Engineering, Hungkuang University, Taichung 433, Taiwan

[8] College of Health and Nursing, Meiho University, Pingtung 912, Taiwan

[9] The iEGG and Animal Biotechnology Center, and the Rong Hsing Research Center for Translational Medicine, National Chung Hsing University, Taichung 402, Taiwan

***Corresponding author**: Chuan-Mu Chen, Ph.D.

Professor and Dean
Department of Life Sciences,
College of Life Sciences,
National Chung Hsing University,
No. 250, Kuo Kuang Road, Taichung 402, Taiwan
Phone: 886-4-22856309; Fax: 886-4-22874740
E-mail: chchen1@dragon.nchu.edu.tw

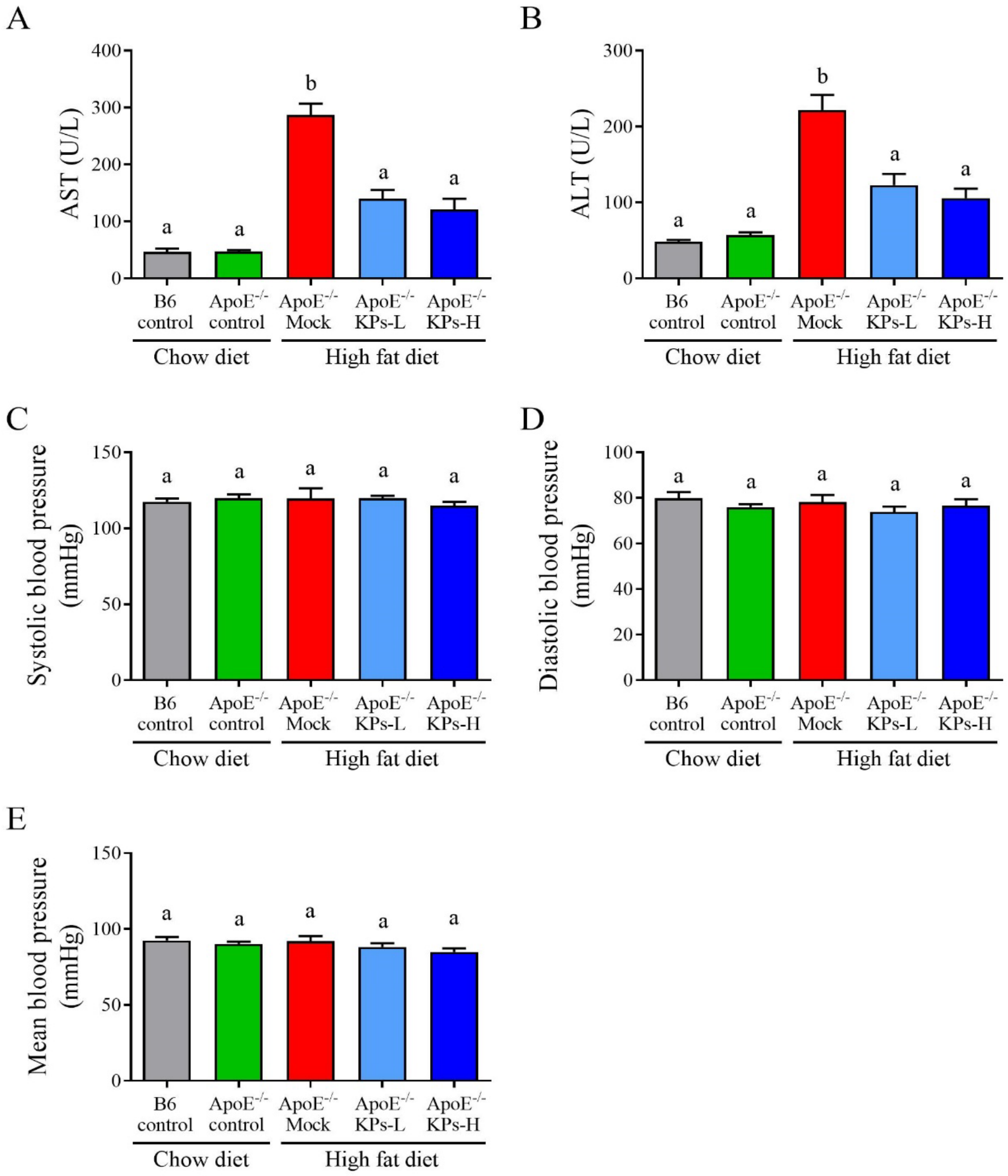

Supplementary Figure 1. Effects of kefir peptides on liver damage markers and blood pressure in HDF-induced atherosclerotic *ApoE$^{-/-}$* mice. (A) Blood aspartate aminotransferase (AST) and (**B**) alanine aminotransferase (ALT) were measured by using a VetTestTM automatic colorimetrically analyzer. **(C)** Systolic blood pressure (SBP), (**D**) diastolic blood pressure (DBP), and (**E**) mean blood pressure (MBP) were measured by the Tail-Cuff detection system (BP-98A, Softron, Tokyo, Japan). Data are displayed as the mean ± SEM (n = 8). The labels at the top of columns without the same letters indicate significant differences between groups ($P < 0.05$).

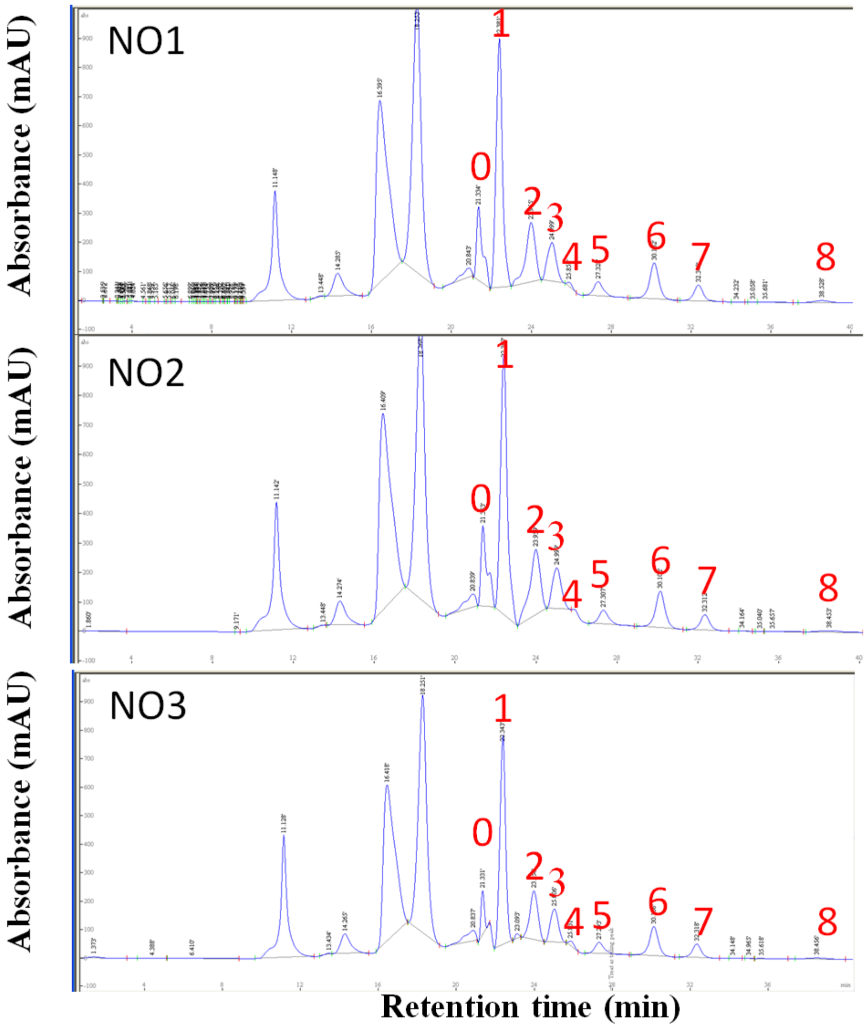

Supplementary Figure 2. The quality controls of kefir peptides powder for the peptides separation and reproducibility. They were separated by semipreparative HPLC on a model PU-980 pump (Jasco, Japan) equipped with a UV detector and a 300 x 7.8 mm i.d., 5-µm particles TSK-GEL G2000SWXL column (Sigma-Aldrich, St Louis, MO). The mobile phase was 100 mM KH2PO4, 1 M NaCl and 1 mM EDTA (pH = 6.5) at a flow rate of 0.5 mL/min, and the wavelength was detected at 215 nm.

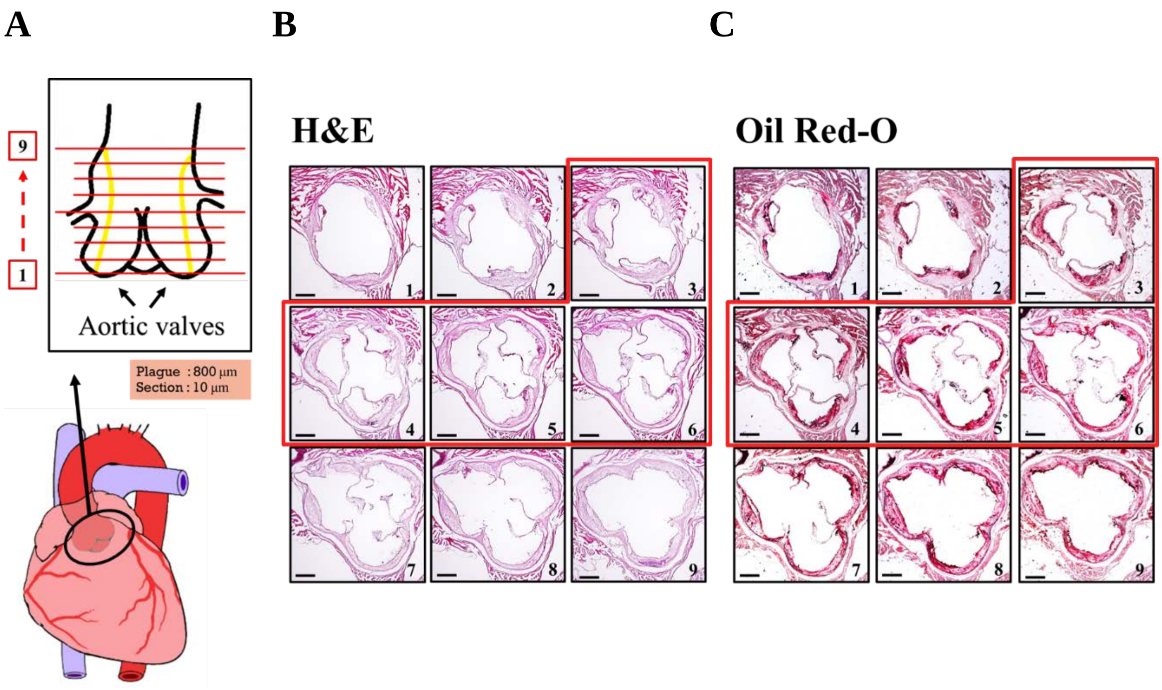

Supplementary Figure 3. Schematic images of aorta continuously serial sections for plaque formation examination in HFD-induced atherosclerotic *ApoE*$^{-/-}$ mic. The accumulation of lipid in the aortic roots of *ApoE*$^{-/-}$ mice was observed by Oil red-O staining and counterstained with H&E. Scale bars: 200 μm. **(A)** The relative dissection position of mouse aortic root in the anatomy view. (**B**) The H&E staining of 9 continuously serial sections from the root to ascending aortic region. (**C**) The Oil red-O staining of 9 continuously serial sections from the root to ascending aortic region. The middle of 4 sections labeled by red box (slides 3-6) were used to quantify the Oil red-O-positive staining area.

Figure 3A. Raw data

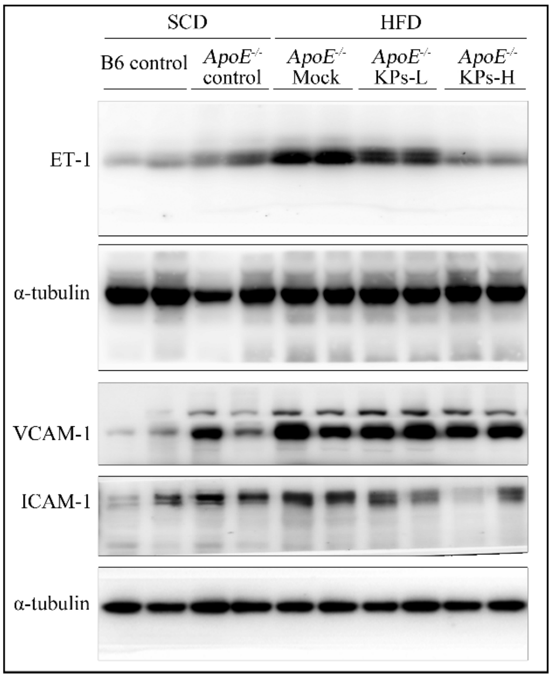

Figure 5E. Raw data

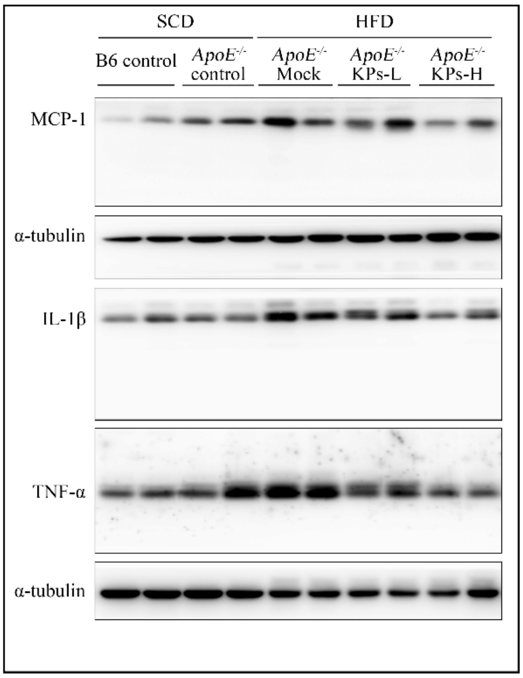

Figure 6D. Raw data

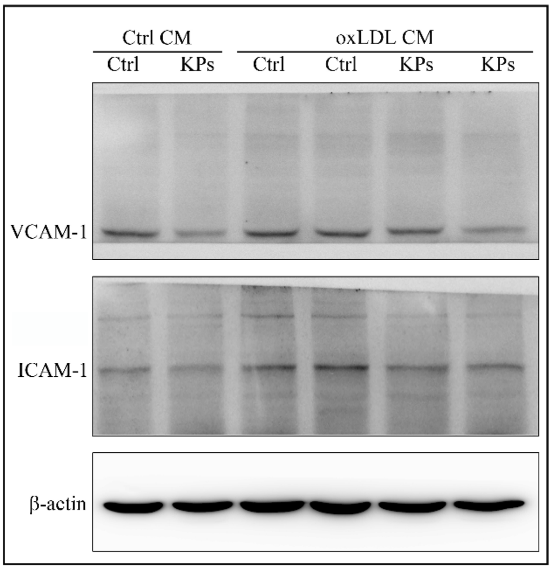

Supplementary Figure 4. Western blot raw data for Fig. 3A, Fig. 5E, and Fig. 6D.
